# Perceived barriers to pre-exposure prophylaxis use among HIV-negative men who have sex with men in Tijuana, Mexico: A latent class analysis

**DOI:** 10.1371/journal.pone.0221558

**Published:** 2019-08-22

**Authors:** Rudy Patrick, Jennifer Jain, Alicia Harvey-Vera, Shirley J. Semple, Gudelia Rangel, Thomas L. Patterson, Heather A. Pines

**Affiliations:** 1 Department of Medicine, University of California San Diego, La Jolla, CA, United States of America; 2 Department of Psychiatry, University of California San Diego, La Jolla, CA, United States of America; 3 US-Mexico Border Health Commission, Tijuana, Mexico; 4 El Colegio de la Frontera Norte, Tijuana, Mexico; University of Washington, UNITED STATES

## Abstract

**Background:**

Given the slow uptake of PrEP among cisgender men who have sex with men (MSM) in high-income countries, efforts to roll-out PrEP in low- and middle-income countries (LMIC) should address barriers to PrEP use to facilitate its more rapid uptake. To inform PrEP programs in LMIC, we examined patterns of perceived barriers to PrEP use among HIV-negative MSM in Tijuana, Mexico.

**Methods:**

From 03/2016-09/2017, 364 MSM completed interviewer-administered surveys assessing perceived barriers to PrEP use across 4 domains: PrEP attribute, individual, interpersonal, and structural. Latent class analysis was performed to identify distinct classes with respect to perceived barriers to PrEP use. Multinomial logistic regression was used to identify factors associated with class membership.

**Results:**

We identified three classes characterized by (1) high levels of perceived barriers across domains (12%), (2) low levels of perceived barriers across domains (43%), and (3) perceived PrEP attribute barriers (i.e., side-effects and cost) (45%). Membership in the high level of perceived barriers class (vs. the low level of perceived barriers class) was positively associated with having a history of incarceration (AOR: 2.44; 95% CI: 1.04, 5.73) and negatively associated with more social support (AOR: 0.99; 95% CI: 0.98, 1.00). Membership in the perceived PrEP attribute barriers class was positively associated with having seen a healthcare provider in the past year (AOR: 2.78; 95% CI: 1.41, 5.45) and negatively associated with having any HIV-positive or status unknown partners (AOR: 0.56; 95% CI: 0.31, 1.01).

**Conclusions:**

Since most participants were in either the low level of perceived barriers class or the perceived PrEP attribute barriers class, future PrEP uptake may be high among MSM in Tijuana. However, these findings suggest that achieving sufficient PrEP uptake and adherence among MSM in Tijuana may require a range of comprehensive HIV prevention interventions.

## Introduction

Worldwide, cisgender men who have sex with men (MSM) are disproportionately impacted by HIV infection [1, 2]. Daily oral use of emtricitabine and tenofovir disoproxil fumarate (FTC/TDF) as pre-exposure prophylaxis (PrEP) effectively prevents HIV infection [3–6] and has the potential to curb HIV epidemics among MSM globally [7–10]. In 2014, the World Health Organization (WHO) released guidelines recommending PrEP for MSM at substantial risk of HIV infection [11]. Yet, to-date, PrEP implementation and uptake have been slow and mainly concentrated among MSM in high-income countries [12, 13].

International research among MSM has identified barriers to PrEP uptake across multiple domains characterized by PrEP attribute, individual, interpersonal, and structural barriers. PrEP attributes are the most commonly cited barriers to PrEP use among MSM, particularly concerns about PrEP’s cost, efficacy, and side-effects [14–20]. Individual barriers to PrEP use include low perceived risk of HIV infection [14, 21–23], concerns about daily adherence [17, 20, 23, 24], and the potential for increased risk of other sexually transmitted infections due to decreased condom use as a result of PrEP use [19, 20]. With respect to interpersonal barriers to PrEP use, prior work has documented concerns that sex partners may expect condomless anal intercourse (CAI) in the context of PrEP use [20], as well as fears that PrEP users may be perceived as sexually promiscuous [21, 25–27] or HIV-positive [18, 24, 28] if others see them taking PrEP. Finally, structural barriers that limit healthcare access can also significantly impact PrEP use. For instance, stigma towards sexual minorities often limits the willingness of MSM to discuss sexual health and HIV prevention with healthcare providers (HCPs) [25–27], which may inhibit their ability to access PrEP [29].

Although many of these barriers to PrEP use have been consistently identified among MSM across diverse settings, less is known about how patterns of perceived barriers may vary across subgroups within MSM populations. Latent class analysis (LCA) identifies unobserved classes or subgroups within populations based on the similarity of their responses across individual measures [30–32]. Although LCA has been used to identify subgroups of MSM with respect to patterns of sexual and substance use risk behaviors, barriers to HIV testing, and HIV risk reduction strategies [33–36], it has not been used to identify subgroups of MSM with respect to patterns of perceived barriers to PrEP use. Understanding the underlying patterns of perceived barriers to PrEP use across subgroups of MSM and whether these subgroups differ by socio-demographic, psychosocial, healthcare access, substance use, and sexual risk factors may help inform future interventions to support PrEP uptake among MSM.

Given that PrEP uptake has been slow among MSM in early adopter countries [13], it is imperative that efforts to bring PrEP to scale in low- and middle-income countries (LMIC), like Mexico, address barriers to PrEP use to support its rapid uptake among those at high risk of HIV acquisition. To inform these efforts, we conducted an exploratory LCA to identify distinct classes of MSM in Tijuana, Mexico with respect to perceived barriers to PrEP use and factors associated with class membership. As in other LMIC, the HIV epidemic in Mexico is concentrated among MSM [37], with HIV prevalence among MSM in Tijuana estimated to be 20% [38]. Several socio-structural factors unique to the Mexico-United States border region, including elevated levels of poverty, sex work, drug trafficking, and migration, contribute to the HIV risk environment among MSM in Tijuana [39, 40]. However, as seen in other LMIC [1, 2], HIV risk and access to HIV prevention services among MSM in Tijuana are also shaped by stigma toward sexual minorities [39–41]. As such, findings from this exploratory study may help inform strategies to ensure that comprehensive HIV prevention packages that include PrEP are accessible to MSM in Mexico and other similar LMIC.

## Methods

### Study population

Between March 2016 and September 2017, 397 participants who tested HIV-negative as part of a parent study, *Proyecto Enlaces* (“Project Links”), were recruited to participate in *Proyecto Redes* (“Project Networks”). The primary aim of *Proyecto Enlaces* was to compare the effectiveness of respondent-driven sampling (RDS) and venue-based sampling (VBS) to identify MSM and persons who identify as transgender women (TW) with previously undiagnosed HIV infection in Tijuana. VBS is a time-space sampling method [42] that was conducted at venues identified via formative research as those where MSM and persons who identify as TW meet sexual partners (e.g., bars, public spaces). RDS is a chain-referral sampling method [43] and was initiated by 32 seeds selected to be diverse with respect to HIV status, age, socio-economic status, sexual orientation, gender identity, and recruitment source (i.e., Tijuana’s government-funded HIV treatment clinic and VBS venues). An additional goal of *Proyecto Enlaces* and *Proyecto Redes* was to characterize the sexual networks of MSM and persons who identify as TW due to the high prevalence of HIV among both populations in Tijuana and their often-overlapping sexual networks [38, 44]. As such, both MSM and persons who identify as TW were recruited via RDS and VBS to participate in these studies.

Eligibility criteria for VBS participants included: no prior HIV diagnosis, cisgender male or transgender female, ≥18 years-old, and anal sex with a cisgender male or transgender female in the past 4 months. Eligible individuals were offered rapid HIV testing (Advanced Quality HIV 1/2 Test Kits, Intec Products, Inc., Xiamen, China) and those who tested HIV-negative were offered enrollment in *Proyecto Redes*. Eligibility criteria for RDS seeds included: cisgender male or transgender female, ≥18 years-old, Tijuana residence, anal sex with a cisgender male or transgender female in the past 4 months, and a social network including ≥15 MSM or persons who identify as TW living in Tijuana. Seeds were given three coupons to recruit MSM peers and peers who identify as TW from their social networks who were then given three coupons to recruit their peers in subsequent recruitment waves. Peer-recruit eligibility criteria were similar to those for seeds, but peer-recruits were not required to live in Tijuana and only had to report anal sex with a cisgender male or transgender female in the past year. Peer-recruits without a prior HIV diagnosis were offered HIV testing. To ensure comparability to VBS participants, peer-recruits who tested HIV-negative and reported anal sex with a cisgender male or transgender female in the past 4 months were offered enrollment in *Proyecto Redes*. Since the *Proyecto Redes* survey only included MSM-specific measures for perceived barriers to PrEP use (described below), the sample for the current study was restricted to 364 HIV-negative MSM (i.e., cisgender males who reported anal sex with other cisgender males in the past 4 months) participants.

### Data collection

Participants completed an interviewer-administered survey assessing socio-demographics, substance use behaviors, psychosocial factors, healthcare access, and perceived barriers to PrEP use. Socio-demographic measures included: age, gender identity, education, employment status, duration of residence in Tijuana, and incarceration history. Frequency of illicit drug use and routes of administration (e.g., injecting) were assessed in the past month for marijuana, methamphetamine, cocaine, heroin, tranquilizers, inhalants, ecstasy, amyl nitrate, barbiturates, gamma-hydroxybutyrate, and ketamine. Alcohol use in the past year was measured using the Alcohol Use Disorders Identification Test (AUDIT), with hazardous alcohol consumption defined as a score ≥8 [45]. With respect to psychosocial factors, participants were asked about their perceived lifetime risk of getting HIV (very likely; likely; neither unlikely nor likely; unlikely; very unlikely). Social support was measured using the 8-item Modified Medical Outcome Study Social Support Survey, which assesses one’s level of social support (e.g., *“If you needed it*, *how often is someone available to turn to for suggestions about how to deal with a personal problem*?*”)* on a scale from 1 (“none of the time”) to 5 (“all of the time”) [46]. Social support scores were computed by averaging responses across items and transforming the resulting average to a score on a 100-point scale; higher scores indicate more social support. Outness about having sex with men was assessed by asking participants to describe their level of “outness” on a scale from 1 (“not out to anyone”) to 7 (“out to everyone”) [47]. Depression was measured using the 10-item Center for Epidemiologic Studies Depression Scale (CESD-10), with depression defined as a score ≥10 [48]. Healthcare access questions assessed whether participants had seen a HCP and been tested for HIV in the past year.

Participants were then given basic information about PrEP, including its effectiveness, the importance of daily adherence, and potential side-effects. To assess awareness and use of PrEP, participants were asked whether they had ever heard of PrEP and, if aware, whether they had ever taken PrEP. To assess willingness to use PrEP, participants were asked how willing they would be to take PrEP if available in Mexico for free and at a cost. Participants who were willing to pay for PrEP, were asked how much they would be willing to spend monthly in Mexican pesos (≤150; 151–350; 351–550; 551–750; 751–950; ≥951). Perceived barriers to PrEP use were assessed by asking participants to indicate their level of agreement with 12 statements about why someone would not want to use PrEP using 5-point Likert scale responses (strongly disagree; disagree; neither agree nor disagree; agree; strongly agree). The statements were developed to assess important barriers to PrEP use identified in previous research [15–17, 20, 23, 49] across four domains: (1) PrEP attributes (e.g., *“PrEP costs too much*), (2) individual (e.g., *“I am at low risk for HV”*), (3) interpersonal (e.g., *“Partners will expect me to have CAI if I take PrEP*), and (4) structural (e.g., *“I will receive poor healthcare if HCPs know I have sex with men”)*.

Finally, participants completed a sexual network survey assessing socio-demographics, HIV status (HIV-negative; HIV-positive; unknown), and sexual and substance use behaviors with ≤20 anal or vaginal sexual partners in the past 4 months. We considered participants’ responses for partners they identified as cisgender male and transgender female (since the likelihood of sexual exposure to HIV is greater with these partners than that with cisgender female partners [37]) to classify participants with respect to whether they had any HIV-positive or status unknown partners, engaged in CAI, used alcohol or illicit drugs before or during sex, and engaged in any transactional sex (i.e., gave or received something of value in exchange for sex) in the past 4 months.

Participants received 450 Mexican pesos for completing the survey and those recruited via RDS received an additional 100 Mexican pesos for each of their peer-recruits that successfully enrolled in the study. All participants provided written informed consent and study procedures were approved by institutional review boards at the University of California, San Diego and Universidad Xochicalco in Tijuana.

### Statistical analysis

We conducted an exploratory LCA to identify distinct classes of participants with respect to perceived barriers to PrEP use. LCA probabilistically assigns individuals with similar response patterns on a series of observed measures to distinct classes of an unobserved latent variable [30–32]. For our LCA, we dichotomized participants’ Likert-scale responses to the 12 items measuring perceived barriers to PrEP use (strongly disagree, disagree, or neither agree nor disagree vs. agree or strongly agree), fit a series of models using those binary indicators with one to five classes, and examined the distribution of the log-likelihood values for each model to determine whether a maximum likelihood (ML) solution could be identified [50]. Although we added Bayesian priors (1.0) to stabilize parameter estimation and fit these models using 100 random starting values to facilitate the identification of an ML solution [51], we were unable to identify an ML solution for models with more than two classes due to the sparseness of our data (2^12^ possible response patterns).

To help facilitate the identification of an ML solution, we considered two different methods of reducing the amount of unknown information [50]. First, we considered all 12 indicators with Bayesian priors (1.0) and imposed parameter restrictions that constrained parameter estimates to be equivalent across classes for two indicators: “*I am concerned about talking to HCPs about having sex with men*” and “*People might assume I am promiscuous*.” These indicators were chosen because they were least prevalent within their respective domains and had similar item-response probabilities within classes to two other indicators (i.e., *“I am concerned about talking to HCPs about having sex with men”* was similar to *“I will receive poor healthcare if HCPs know I have sex with men”* and *“People might assume I am promiscuous”* was similar to *“People might assume I am HIV+”*) [51]. Second, we considered only 10 indicators, excluding *“I am concerned about talking to HCPs about having sex with men”* and *“People might assume I am promiscuous*.*”* Using the first method we were again unable to identify an ML solution for models with more than two classes. However, using the second method, we were able to identify models with one to three classes. Therefore, we proceeded with the models identified via the second method. Next, we examined bivariate residuals (BVRs) to determine whether the local independence assumption underlying LCA may be violated (S1 Table) and allowed for local dependence between pairs of indicators with statistically significant associations (i.e., BVR>3.84) [51, 52]. To select our final model, we considered fit statistics (Akaike’s information criteria [AIC], Bayesian information criteria [BIC], sample size-adjusted Bayesian information criteria [a-BIC]) and entropy as a measure of classification certainty. Lower AIC and BIC values and higher values of entropy were considered indicators of better model fit. In addition, we examined class separation (e.g., distinct patterns of item-response probabilities across latent classes), within-class homogeneity (e.g., similarity of item-response probability patterns within classes), and the meaningfulness of identified classes [50, 51].

We then examined whether class membership was associated with socio-demographic, substance use, psychosocial, healthcare access, and sexual risk factors using Vermunt’s bias-adjusted three-step approach [53]. First, we used the posterior probabilities of class membership estimated from our final model (selected in Step 1) to assign participants to one of three classes based on the proportional assignment method (Step 2). Next, we used multinomial logistic regression to examine the relationship between class membership and the factors of interest (Step 3) applying Vermunt’s ML method to correct for classification errors in Step 2 and a robust variance estimator [53, 54]. Based on previous research examining factors associated with barriers to PrEP use and willingness to use PrEP among MSM [15, 21, 26, 55], we created directed acyclic graphs (DAGs) [56] to illustrate potential relationships among the factors of interest and class membership. We then used these DAGs to identify confounders of the relationship between each factor of interest and class membership. Finally, we constructed individual models for each factor to account for the possibility that a confounder of the relationship between one factor and class membership could be a mediator of the relationship between another factor and class membership [57, 58]. Data management and descriptive analyses were conducted using SAS 9.4 [59] and the LCA was conducted using Latent GOLD 5.1 [51].

## Results

### Sample characteristics

Our sample (N = 364) had a mean age of 38.3 years (SD: 11.5), mostly identified as Hispanic (99%), and approximately half reported at least a high school education (42%) and having seen a HCP in the past year (51%) (Table 1). Despite low prior PrEP awareness (17%) and use (5%), 88% were willing to take PrEP if it was available for free. Of participants willing to pay for PrEP (60%), 30% reported that they would only be willing to pay for it if it cost ≤150 Mexican pesos (~8.0 US dollars).

**Table 1:** Socio-demographic, substance use, sexual risk, psychosocial, and healthcare access characteristics among MSM in Tijuana, Mexico (N = 364).

	n (%)
**Recruitment Method**	
RDS	187 (51.4)
VBS	177 (48.6)
**Socio-demographics**	
Median age (years)	39 (IQR: 28.5–46.5)
Age (years)	
18–24	49 (13.5)
25–29	53 (14.6)
30–39	84 (23.1)
≥40	178 (48.9)
Hispanic ethnicity	360 (98.9)
Gay identifying	128 (35.2)
≥ High school education	151 (41.5)
Employed	220 (60.4)
Median duration of residence in Tijuana (years)	9 (IQR: 3–21)
Incarceration history	157 (43.1)
**Psychosocial factors**	
Social support, mean score	56.3 (SD: 34.8)
Outness about having sex with men, mean score	4.0 (SD: 2.5)
Depression (CESD-D ≥10)	163 (44.8)
**Substance use**	
Illicit drug use (≤1 month)^a^	189 (51.9)
Marijuana	128 (35.2)
Methamphetamine	159 (43.7)
Cocaine	45 (12.4)
Heroin	62 (17.0)
Tranquilizers	52 (14.3)
Inhalants	9 (2.5)
Ecstasy	20 (5.5)
Amyl nitrite (poppers)	26 (7.1)
Barbiturates	15 (4.1)
GHB	8 (2.2)
Ketamine	5 (1.4)
Injection drug use	63 (17.3)
Hazardous alcohol consumption (≤12 months)	196 (53.9)
**Sexual Risk Behaviors (past 4 months)**	
Any HIV-positive/status unknown partners	158 (43.4)
Any substance use before/during sex with partners	266 (73.1)
Gave/received something of value to/from partners in exchange for sex	157 (43.1)
Any CAI with partners	251 (69.2)
**Healthcare Access**	
Received care from a HCP, past 12 months	186 (51.1)
HIV testing (≤12 months)	161 (44.2)
PrEP awareness	62 (17.0)
PrEP use (n = 62)	3 (4.8)
Willing to use PrEP if available for free	319 (87.6)
Willing to use PrEP if available, but not for free	217 (59.6)
If PrEP were available, but not for free, how much would be willing to pay per month (pesos) (n = 217):^b^	
≤150	64 (29.5)
150–350	40 (18.4)
351–550	46 (21.2)
551–750	23 (10.6)
751–950	11 (5.1)
950+	33 (15.2)
Perceived risk of HIV (likely/very likely)	179 (49.2)

Abbreviations: CESD-D = center for epidemiologic studies depression scale; MSM = men who have sex with men; RDS = respondent-driven sampling; VBS = venue-based sampling; HCP = healthcare provider; CAI = condomless anal intercourse; PrEP = pre-exposure prophylaxis; SD = standard deviation; IQR = interquartile range.

^a^ Excluding marijuana.

^b^ 2016–2017 conversion rate: 1 US dollar = 18.74 Mexican pesos.

### Perceived barriers to PrEP use

Perceived barriers to PrEP use in the PrEP attribute domain were most common: “*PrEP costs too much*” (50%) and “*I am concerned about long-term side-effects*” (41%) (Table 2). Followed by “*I have limited access to healthcare services*” (32%) in the structural domain, “*Taking PrEP might tempt me to have CAI*” (31%) in the individual domain, and “*People might assume I am HIV+*” (28%) in the interpersonal domain.

**Table 2:** Perceived barriers to PrEP use among MSM in Tijuana, Mexico (N = 364).

Barriers	Proportion Agree/ Strongly Agree
***Product Attributes Domain***	*** ***
PrEP costs too much	50.0
I am concerned about long-term side effects	40.6
PrEP does not fully protect against HIV	20.3
***Individual Domain***	*** ***
I am at low risk for HIV/AIDS	23.6
I would have trouble taking PrEP daily	26.9
Taking PrEP might tempt me to have CAI	31.0
***Interpersonal Domain***	*** ***
Partners will expect me to have CAI if I take PrEP	26.4
People might assume I am HIV+	27.8
People might assume I am promiscuous	26.1
***Structural Domain***	*** ***
I have limited access to healthcare services	31.6
I am concerned about talking to HCP about having sex with men	15.1
I will receive poor healthcare if HCP knows I have sex with men	16.5

Abbreviations: CAI = condomless anal intercourse; HCP = healthcare provider; PrEP = pre-exposure prophylaxis

### Latent classes of perceived barriers to PrEP use

Of the three identified models, the AIC and BIC values were lowest for the three-class model (Table 3), suggesting that it fit the data best. However, because BVRs from all three models indicated a violation of the local independence assumption (S1 Table), we re-fit the two- and three-class models with direct effects between indicators with BVRs >3.84 to account for the relationships between those indicators, which improved the AIC and BIC values for both models relative to those without direct effects. We then considered item-response probabilities from the two models with direct effects and found that the three-class model had better within class homogeneity and between class separation relative to the two-class model and also identified more meaningful classes than the two-class model (S2 Table). Thus, we selected the three-class model with direct effects as our final model.

**Table 3:** Fit statistics across LCA models with an ML solution.

LCA Model	Log Likelihood	Likelihood Ratio Test	AIC	a-BIC	Entropy	DF	X^2^
1-class	-2132.06	1308.31	4284.11	4291.36	1.00	354.00	105778.53
2-class	-1809.32	662.83	3660.63	3675.85	0.83	343.00	1405.15
3-class	-1756.60	557.41	3577.21	3600.40	0.77	332.00	1266.21
2-class DE	-1749.36	542.91	3558.71	3580.45	0.74	334.00	1287.97
3-class DE	-1735.44	515.07	3540.87	3566.23	0.74	329.00	1180.03

Abbreviations: AIC = Akaike’s information criteria; BIC = Bayesian information criteria; a-BIC = sample-size adjusted Bayesian information criteria; DE = direct effects; DF = degrees of freedom; LCA = latent class analysis; ML = maximum likelihood.

**Fig 1:**
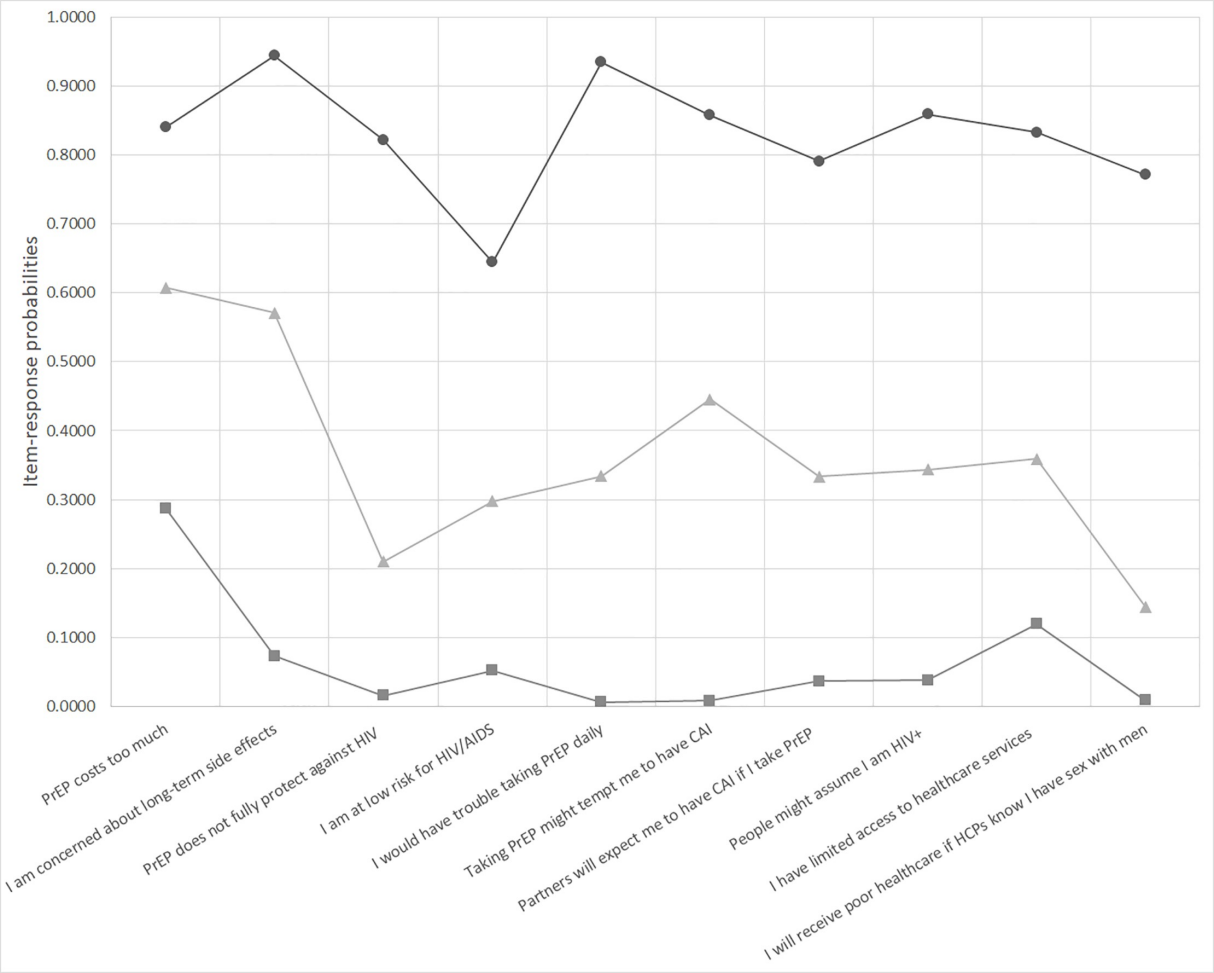
Item-response probabilities across classes of MSM in Tijuana, Mexico with varying patterns of perceived barriers to PrEP use as identified by Latent Class Analysis (N = 364). Identified classes consist of MSM with high-levels of perceived barriers (circle; 12.0%), low-levels of perceived barriers (square; 43.0%), and those who perceived PrEP attribute barriers (triangle; 45.0%). Abbreviations: CAI = condomless anal intercourse; HCP = healthcare provider; MSM = men who have sex with men; PrEP = pre-exposure prophylaxis.

Based on the item-response probabilities for each of the perceived barriers (i.e., indicators) across classes identified by our final model (S2 Table; Fig 1), we labelled the classes (1) high level of perceived barriers (12%), (2) low level of perceived barriers (43%), and (3) perceived PrEP attribute barriers (45%). Participants in the “high level of perceived barriers” class had a high probability (0.64–0.94) of perceiving all of the measured barriers as barriers to PrEP use, while those in the “low level of perceived barriers” class had a low probability (0.007–0.288) of perceiving any of the measured barriers as barriers to PrEP use. Participants in the “perceived PrEP attribute barriers” class had a moderately high probability of perceiving cost (0.61) and side-effects (0.57) as barriers to PrEP use.

### Factors associated with latent class membership

In multivariable analyses (Table 4), membership in the “high level of perceived barriers” class (vs. the “low level of perceived barriers” class) was negatively associated with greater social support (AOR: 0.99; 95% CI: 0.98–1.00) and positively associated with a history of incarceration (AOR: 2.44, 95% CI: 1.04–5.73). Membership in the “perceived PrEP attribute barriers” class, compared to the “low level of perceived barriers” class, was positively associated with receiving care from a HCP in the past year (AOR: 2.78; 95% CI: 1.41, 5.45) and negatively associated with having any HIV-positive or status unknown partners (AOR: 0.56; 95% CI: 0.31, 1.01).

**Table 4:** Socio-demographic, substance use, sexual risk, psychosocial, and healthcare access characteristics associated with membership in distinct classes with respect to perceived barriers to PrEP use among MSM in Tijuana, Mexico (N = 364).

	Perceived PrEP Attribute Barriers				High Level of Perceived Barriers			
	OR	95% CI	AOR	95% CI	OR	95% CI	AOR	95% CI
Socio-demographics								
Age (years)	0.99	(0.97, 1.01)	0.99	(0.97, 1.01)^b^	1.01	(0.99, 1.04)	1.01	(0.99, 1.04)^b^
≥ High school education	1.20	(0.71, 2.03)	1.15	(0.67, 1.96)^c^	0.41	(0.17, 0.96)	0.43	(0.18, 1.02)^c^
Duration of residence in Tijuana (years)	1.00	(0.98, 1.02)	1.00	(0.98, 1.02)^d^	0.98	(0.95, 1.01)	0.98	(0.96, 1.01)^d^
Incarceration history	0.95	(0.55, 1.62)	1.08	(0.60, 1.95)^e^	2.58	(1.22, 5.47)	2.44	(1.04, 5.73)^e^
Employed	1.28	(0.74, 2.20)	1.25	(0.71, 2.19)^f^	0.50	(0.24, 1.03)	0.68	(0.30, 1.53)^f^
Psychosocial factors								
Social support	1.00	(0.99, 1.01)	1.00	(0.99, 1.01)^g^	0.99	(0.98, 1.00)	0.99	(0.98, 1.00)^g^
Outness about having sex with men	0.95	(0.86, 1.06)	0.94	(0.84, 1.05)^h^	0.79	(0.66, 0.93)	0.84	(0.69, 1.02)^h^
Depression (CESD-D ≥10)	0.95	(0.56, 1.62)	0.94	(0.54, 1.65)^i^	1.77	(0.86, 3.66)	1.10	(0.49, 2.45)^i^
Substance use								
Illicit drug use (≤1 month)^a^	1.12	(0.66, 1.88)	1.09	(0.58, 2.06)^j^	2.42	(1.13, 5.19)	1.57	(0.61, 4.01)^j^
Hazardous alcohol consumption (≤12 months)	0.65	(0.38, 1.10)	0.62	(0.35, 1.08)^j^	0.75	(0.37, 1.55)	0.66	(0.30, 1.43)^j^
Sexual Risk Behaviors (past 4 months)								
Any HIV-positive/status unknown partners	0.58	(0.34, 0.99)	0.56	(0.31, 1.01)^k^	0.97	(0.47, 1.99)	0.91	(0.40, 2.05)^k^
Any substance use before/during sex with partners	0.75	(0.42, 1.34)	0.85	(0.42, 1.74)^k^	1.36	(0.57, 1.67)	0.76	(0.20, 2.82)^k^
Gave/received something of value to/from partners in exchange for sex	0.98	(0.58, 1.67)	0.95	(0.53, 1.72)^k^	3.02	(1.41, 6.48)	1.96	(0.76, 5.04)^k^
Any CAI with partners	0.86	(0.49, 1.51)	0.91	(0.49, 1.66)^l^	1.64	(0.71, 3.81)	1.87	(0.54, 6.44)^l^
Healthcare Access								
Received care from a HCP (≤12 months)	2.07	(1.21, 3.53)	2.78	(1.41, 5.45)^m^	0.72	(0.34, 1.52)	1.26	(0.49, 3.23)^m^
HIV testing (≤12 months)	1.67	(0.99, 2.84)	1.62	(0.91, 2.89)^m^	0.78	(0.36, 1.68)	1.14	(0.48, 2.72)^m^
PrEP awareness	1.05	(0.54, 2.03)	0.82	(0.37, 1.81)^n^	0.36	(0.08, 1.57)	0.44	(0.03, 5.67)^n^
Perceived lifetime risk of HIV (likely/very likely)	0.88	(0.52, 1.48)	0.98	(0.52, 1.82)^o^	1.10	(0.54, 2.26)	0.85	(0.35, 2.03)^o^

^a^ Excluding marijuana.

^b^ No identified confounders.

^c^ Adjusted for age.

^d^ Adjusted for variable in (c); additionally, adjusted for education.

^e^ Adjusted for variables in (c) and (d); additionally, adjusted for duration of residence in Tijuana.

^f^ Adjusted for variables in (c), (d), and (e); additionally, adjusted for incarceration history.

^g^ Adjusted for variables in (c), (d), (e), and (f); additionally, adjusted for employment.

^h^ Adjusted for variables in (c), (d), (e), (f), and (g); additionally, adjusted for social support.

^i^ Adjusted for variables in (c), (d), (e), (f), (g), and (h); additionally, adjusted for outness.

^j^ Adjusted for variables in (c), (d), (e), (f), (g), (h), and (i); additionally, adjusted for depression.

^k^ Adjusted for variables in (c), (d), (e), (f), (g), (h), (i), and (j); additionally, adjusted for illicit drug use and hazardous alcohol consumption.

^l^ Adjusted for variables in (c), (d), (e), (f), (g), (h), (i), (j), and (k); additionally, adjusted for HIV status of partners, substance use before/during sex with partners, and

exchange of something of value for sex with partners.

^m^ Adjusted for variables in (c), (d), (e), (f), (g), (h), (i), (j), (k), and (l); additionally, adjusted for any CAI.

^n^ Adjusted for variables in (c), (d), (e), (f), (g), (h), (i), (j), (k), (l), and (m); additionally, adjusted for receipt of care from a HCP and HIV testing.

^o^ Adjusted for variables in (c), (d), (e), (f), (g), (h), (i), (j), (k), (l), (m), and (n); additionally, adjusted for PrEP awareness.

Abbreviations: CESD-D = center for epidemiologic studies depression scale; MSM = men who have sex with men; HCP = healthcare provider; CAI = condomless anal intercourse;

PrEP = pre-exposure prophylaxis.

Reference group = low level of perceived barriers.

## Discussion

We assessed perceived barriers to PrEP use across four domains (PrEP attribute, individual, interpersonal, and structural) among HIV-negative MSM in Tijuana, Mexico. Using exploratory LCA, we identified three classes of participants with respect to patterns of perceived barriers to PrEP use. It is promising for future PrEP implementation that 43% of participants endorsed few barriers to PrEP use. However, 45% of participants perceived PrEP attributes, including cost and side-effects, as barriers to PrEP use, while 12% perceived multiple barriers to PrEP use. Although our LCA was exploratory, our findings provide important insight on how PrEP programs might be developed to best serve the needs of MSM in Tijuana. More specifically, our results suggest that a range of comprehensive HIV prevention packages may be needed to enhance PrEP uptake and adherence across subgroups of MSM in this setting.

Consistent with prior research among MSM in LMIC [16, 18, 55], PrEP attributes were commonly perceived barriers to PrEP use among participants in both the “perceived PrEP attribute barriers” and “high level of perceived barriers” classes. Although antiretroviral therapy (ART) is universally available in Mexico [60], it is currently unknown how PrEP will be incorporated into Mexico’s healthcare system. Branded FTC/TDF can cost up to 850 US dollars per month in high-income countries [61], while generic FTC/TDF, which is used as ART in some LMIC [61, 62], is estimated to cost 6.5 US dollars per month (~123 Mexican pesos). In our study, 30% of participants who reported that they would be willing to pay for PrEP said they would only do so if it cost ≤150 Mexican pesos (~8.0 US dollars) per month. Thus, making generic FTC/TDF available as PrEP in Mexico may enhance its acceptability to many MSM in Tijuana, while its integration into Mexico’s public healthcare system may fully address concerns related to cost within this vulnerable population. Although PrEP use has been associated with several mild, short-term side-effects [63], and non-progressive and reversible declines in renal function and bone density [64], it is generally considered safe and well tolerated [65]. Given that almost half (45%) of our sample was in the “perceived PrEP attribute barriers” class, raising potential PrEP users’ awareness of cost coverage options that may enhance its affordability and increasing their knowledge of the nature and duration of potential side-effects may significantly improve PrEP uptake among MSM in Tijuana.

It is promising that MSM with any HIV-positive or status unknown partners were less likely to be in the “perceived PrEP attribute barriers” class, indicating those who could benefit the most from PrEP may experience few barriers to PrEP uptake. Additionally, we found that having seen a HCP in the past year was associated with membership in this class. MSM already engaged in healthcare may perceive themselves to be healthy and thus at low risk for HIV infection. As such, they may feel the additional cost and potential side-effects of PrEP use are not worth the benefits. This finding suggests that achieving an increased level of PrEP coverage may be easily addressed in the context of provider-delivered PrEP education. However, the impact of such education on PrEP uptake may also depend on HCPs’ knowledge and perceptions of PrEP, which previous research suggests is linked to their willingness to prescribe PrEP [66, 67]. There is also evidence that heterosexism may decrease HCPs’ willingness to prescribe PrEP to MSM [68, 69], which could adversely affect PrEP provision to sexual minorities in Tijuana where they remain highly stigmatized. Thus, efforts to raise HCPs’ awareness of the benefits of PrEP and bolster their capacity to deliver culturally competent PrEP-related care to their sexual minority patients, including patient-centered, non-judgmental sexual risk assessment, may be critical to the success of provider-delivered PrEP education in Tijuana.

However, to achieve complete PrEP coverage among MSM in Tijuana, additional efforts may be needed to address the range of barriers to PrEP use endorsed by those in the “high level of perceived barriers” class within our sample. For example, a history of incarceration was associated with membership in this class. Persons with a history of incarceration often face elevated rates of unemployment, substance use, mental illness, and inadequate access to healthcare [70–72], which may limit PrEP uptake among this population. A peer navigation intervention initiated pre-release, which addressed social-environmental factors and incorporated individualized problem-solving techniques, was shown to effectively support viral suppression maintenance among HIV-positive men and TW released from jail [73]. Interventions which assess PrEP interest and eligibility during incarceration and provide peer navigation services post-release may support PrEP uptake and adherence and be particularly effective for MSM like those in the “high level of perceived barriers” class. However, lower levels of social support were also associated with being in this class. In Mexico, cultural norms of homophobia and *machismo* often inhibit MSM from being able to “come out” and positively experience their sexual identity [74]. This can cause psychological distress among MSM, resulting in withdrawal from social support networks (e.g., family and friends) [75, 76], which can impact their ability to access culturally competent healthcare [41, 74, 77]. As such, community-level programs which reduce negative social attitudes towards MSM in combination with counseling interventions to help MSM identify supportive social network members may be critical to PrEP use and adherence among MSM in Tijuana.

Our findings should be considered in the context of several limitations. First, our analysis was based on cross-sectional data, which limits our ability to make causal inferences. Second, participants were recruited using non-probability sampling methods, thus our findings may not be generalizable to all MSM in Tijuana or other settings. However, MSM experience significant levels of stigma and discrimination related to being sexual minorities, therefore using VBS and RDS may have increased our ability to access this hard-to-reach population. Third, this study is based on self-reported data, which may have been under-reported due to recall or social desirability bias. Participants may have also overstated their willingness to use PrEP given interviewers had just informed them of its potential benefits. However, interviewers were trained to encourage honest reporting of sensitive information. Fourth, we report on perceived barriers to PrEP use among MSM in Tijuana, which may differ from the actual barriers they may face once PrEP is implemented in Tijuana. Finally, because we were unable to identify ML solutions for models with more than three classes, our findings should be examined further in future research with a larger sample of MSM in the context of a confirmatory LCA.

## Conclusion

Despite these limitations, our study is the first to examine perceived barriers to PrEP use among MSM in Tijuana. These findings, while exploratory, suggest that subgroups with respect to perceived barriers do exist among MSM in Tijuana and provide insight on the range of comprehensive HIV prevention packages that may be needed to achieve sufficient PrEP coverage within this vulnerable population.

## Supporting information

S1 Table Bivariate residuals for models with 10 indicators and Bayesian priors. (DOCX)

S2 Table Item-response probabilities for models with ML solutions. (DOCX)

S1 File Data used for this paper. (CSV)

S2 File Codebook for analysis. (CSV)
