# Supplementary material for: Perceived barriers to pre-exposure prophylaxis use among HIV-negative men who have sex with men in Tijuana, Mexico: A latent class analysis
Source: PLoS One. 2019 Aug 22;14(8):e0221558. doi: 10.1371/journal.pone.0221558 (PMC6705824; doi:10.1371/journal.pone.0221558)
Supplement: S1 Table — (DOCX) [file pone.0221558.s001.docx]

| **S1 Table. Bivariate residuals for models with 10 indicators and Bayesian priors.** | | | | | | | | | | | |
| --- | --- | --- | --- | --- | --- | --- | --- | --- | --- | --- | --- |
| **LCA Model** | **Indicators** | **PrEP costs too much** | **I am concerned about long-term side effects** | **PrEP does not fully protect against HIV** | **I am at low risk for HIV/AIDS** | **I would have trouble taking PrEP daily** | **Taking PrEP might tempt me to have CAI** | **Partners will expect me to have CAI if I take PrEP** | **People might assume I am HIV+** | **I have limited access to healthcare services** | **I will receive poor healthcare if HCP knows I have sex with men** |
| **1-Class** | **PrEP costs too much** | . |  |  |  |  |  |  |  |  |  |
|  | **I am concerned about long-term side effects** | 21.12 | . |  |  |  |  |  |  |  |  |
|  | **PrEP does not fully protect against HIV** | 15.22 | 40.29 | . |  |  |  |  |  |  |  |
|  | **I am at low risk for HIV/AIDS** | 24.29 | 39.66 | 70.98 | . |  |  |  |  |  |  |
|  | **I would have trouble taking PrEP daily** | 20.11 | 75.84 | 67.78 | 47.64 | . |  |  |  |  |  |
|  | **Taking PrEP might tempt me to have CAI** | 15.68 | 51.50 | 57.73 | 21.23 | 69.04 | . |  |  |  |  |
|  | **Partners will expect me to have CAI if I take PrEP** | 9.54 | 36.69 | 44.04 | 20.82 | 41.84 | 117.37 | . |  |  |  |
|  | **People might assume I am HIV+** | 18.71 | 38.35 | 59.11 | 27.74 | 53.71 | 85.73 | 88.01 | . |  |  |
|  | **I have limited access to healthcare services** | 25.67 | 24.95 | 33.28 | 22.33 | 28.52 | 29.45 | 33.55 | 33.72 | . |  |
|  | **I will receive poor healthcare if HCP knows I have sex with men** | 13.45 | 35.18 | 58.41 | 15.42 | 52.80 | 46.54 | 55.05 | 54.13 | 40.78 | . |
| **2-Class** | **PrEP costs too much** | . |  |  |  |  |  |  |  |  |  |
|  | **I am concerned about long-term side effects** | 2.83 | . |  |  |  |  |  |  |  |  |
|  | **PrEP does not fully protect against HIV** | 0.53 | 0.17 | . |  |  |  |  |  |  |  |
|  | **I am at low risk for HIV/AIDS** | 6.30 | 2.99 | 9.55 | . |  |  |  |  |  |  |
|  | **I would have trouble taking PrEP daily** | 1.47 | 7.16 | 2.03 | 2.61 | . |  |  |  |  |  |
|  | **Taking PrEP might tempt me to have CAI** | 0.11 | 0.20 | 0.05 | 1.11 | 0.49 | . |  |  |  |  |
|  | **Partners will expect me to have CAI if I take PrEP** | 0.02 | 0.01 | 0.01 | 0.19 | 0.07 | 12.03 | . |  |  |  |
|  | **People might assume I am HIV+** | 0.73 | 0.11 | 0.30 | 0.04 | 0.00 | 1.51 | 4.92 | . |  |  |
|  | **I have limited access to healthcare services** | 8.65 | 1.00 | 1.76 | 1.67 | 0.63 | 0.21 | 1.86 | 0.99 | . |  |
|  | **I will receive poor healthcare if HCP knows I have sex with men** | 1.38 | 1.62 | 4.63 | 0.00 | 3.23 | 0.92 | 4.31 | 2.62 | 7.66 | . |
| **3-Class** | **PrEP costs too much** | . |  |  |  |  |  |  |  |  |  |
|  | **I am concerned about long-term side effects** | 0.05 | . |  |  |  |  |  |  |  |  |
|  | **PrEP does not fully protect against HIV** | 0.07 | 0.04 | . |  |  |  |  |  |  |  |
|  | **I am at low risk for HIV/AIDS** | 2.49 | 0.68 | 6.94 | . |  |  |  |  |  |  |
|  | **I would have trouble taking PrEP daily** | 0.00 | 2.62 | 0.43 | 0.91 | . |  |  |  |  |  |
|  | **Taking PrEP might tempt me to have CAI** | 0.92 | 0.63 | 0.01 | 2.38 | 0.02 | . |  |  |  |  |
|  | **Partners will expect me to have CAI if I take PrEP** | 1.98 | 1.27 | 0.23 | 1.15 | 1.26 | 8.45 | . |  |  |  |
|  | **People might assume I am HIV+** | 0.00 | 0.79 | 0.22 | 0.16 | 0.09 | 1.86 | 4.03 | . |  |  |
|  | **I have limited access to healthcare services** | 3.80 | 0.00 | 0.02 | 0.23 | 0.17 | 0.08 | 0.11 | 0.09 | . |  |
|  | **I will receive poor healthcare if HCP knows I have sex with men** | 0.05 | 0.01 | 0.14 | 1.41 | 0.00 | 0.03 | 0.37 | 0.22 | 0.71 | . |

Abbreviations: CAI=condomless anal intercourse; HCP=healthcare provider; LCA=latent class analysis; PrEP=pre-exposure prophylaxis
