# Supplementary material for: Perceived barriers to pre-exposure prophylaxis use among HIV-negative men who have sex with men in Tijuana, Mexico: A latent class analysis
Source: PLoS One. 2019 Aug 22;14(8):e0221558. doi: 10.1371/journal.pone.0221558 (PMC6705824; doi:10.1371/journal.pone.0221558)
Supplement: S2 Table — (DOCX) [file pone.0221558.s002.docx]

| **S2 Table. Item-response probabilities for models with ML solutions.** | | | | | | | | | | | | |
| --- | --- | --- | --- | --- | --- | --- | --- | --- | --- | --- | --- | --- |
|  | **Model 1** | **Model 2** | | **Model 3** | | | **Model 2 DE** | | **Model 3 DE** | | |  |
| *Latent Class* | 1 | 1 | 2 | 1 | 2 | 3 | 1 | 2 | 1 | 2 | 3 |  |
| *Latent Class Prevalences* | 1.00 | 0.67 | 0.33 | 0.48 | 0.40 | 0.13 | 0.62 | 0.38 | 0.45 | 0.43 | 0.12 |  |
| *Item-response probabilities corresponding to "Agree/Strongly Agree" response* |  |  |  |  |  |  |  |  |  |  |  |  |
| PrEP costs too much | 0.5000 | 0.3988 | 0.7043 | 0.3211 | 0.6049 | 0.8516 | 0.3774 | 0.6967 | 0.6075 | 0.2877 | 0.8403 |  |
| I am concerned about long-term side effects | 0.4061 | 0.2203 | 0.7781 | 0.1085 | 0.5985 | 0.9238 | 0.1876 | 0.7537 | 0.5710 | 0.0732 | 0.9441 |  |
| PrEP does not fully protect against HIV | 0.2033 | 0.0378 | 0.5373 | 0.0194 | 0.2338 | 0.8085 | 0.0297 | 0.4820 | 0.2099 | 0.0158 | 0.8219 |  |
| I am at low risk for HIV/AIDS | 0.2363 | 0.1016 | 0.5081 | 0.0538 | 0.3214 | 0.6637 | 0.0877 | 0.4747 | 0.2981 | 0.0521 | 0.6445 |  |
| I would have trouble taking PrEP daily | 0.2692 | 0.0810 | 0.6492 | 0.0282 | 0.3590 | 0.9053 | 0.0495 | 0.6219 | 0.3343 | 0.0066 | 0.9345 |  |
| Taking PrEP might tempt me to have CAI | 0.3104 | 0.0950 | 0.7452 | 0.0102 | 0.4876 | 0.8969 | 0.0686 | 0.6986 | 0.4457 | 0.0087 | 0.8574 |  |
| Partners will expect me to have CAI if I take PrEP | 0.2637 | 0.0825 | 0.6295 | 0.0193 | 0.3780 | 0.8357 | 0.1050 | 0.5186 | 0.3335 | 0.0366 | 0.7905 |  |
| People might assume I am HIV+ | 0.2775 | 0.0765 | 0.6831 | 0.0361 | 0.3792 | 0.8770 | 0.0714 | 0.6081 | 0.3434 | 0.0385 | 0.8589 |  |
| I have limited access to healthcare services | 0.3159 | 0.1874 | 0.5753 | 0.1543 | 0.3415 | 0.8516 | 0.1854 | 0.5254 | 0.3592 | 0.1196 | 0.8325 |  |
| I will receive poor healthcare if HCPs know I have sex with men | 0.1648 | 0.0442 | 0.4084 | 0.0197 | 0.1577 | 0.7410 | 0.0503 | 0.3486 | 0.1446 | 0.0090 | 0.7711 |  |
| Abbreviations: CAI=condomless anal intercourse; DE=direct effects; HCP=healthcare provider; ML=maximum likelihood; PrEP=pre-exposure prophylaxis. | | | | | | | | | | | | |
